# High Prevalence and Spatial Distribution of *Strongyloides stercoralis* in Rural Cambodia

**DOI:** 10.1371/journal.pntd.0002854

**Published:** 2014-06-12

**Authors:** Virak Khieu, Fabian Schär, Armelle Forrer, Jan Hattendorf, Hanspeter Marti, Socheat Duong, Penelope Vounatsou, Sinuon Muth, Peter Odermatt

**Affiliations:** 1 National Centre for Parasitology, Entomology and Malaria Control, Ministry of Health, Phnom Penh, Cambodia; 2 Department of Epidemiology and Public Health, Swiss Tropical and Public Health Institute, Basel, Switzerland; 3 University of Basel, Basel, Switzerland; 4 Medical Department and Diagnostics, Swiss Tropical and Public Health Institute, Basel, Switzerland; S. Cuore Hospital, Italy

## Abstract

**Background:**

The threadworm, *Strongyloides stercoralis*, endemic in tropical and temperate climates, is a neglected tropical disease. Its diagnosis requires specific methods, and accurate information on its geographic distribution and global burden are lacking. We predicted prevalence, using Bayesian geostatistical modeling, and determined risk factors in northern Cambodia.

**Methods:**

From February to June 2010, we performed a cross-sectional study among 2,396 participants from 60 villages in Preah Vihear Province, northern Cambodia. Two stool specimens per participant were examined using Koga agar plate culture and the Baermann method for detecting *S. stercoralis* infection. Environmental data was linked to parasitological and questionnaire data by location. Bayesian mixed logistic models were used to explore the spatial correlation of *S. stercoralis* infection risk. Bayesian Kriging was employed to predict risk at non-surveyed locations.

**Principal Findings:**

Of the 2,396 participants, 44.7% were infected with *S. stercoralis*. Of 1,071 strongyloidiasis cases, 339 (31.6%) were among schoolchildren and 425 (39.7%) were found in individuals under 16 years. The incidence of *S. stercoralis* infection statistically increased with age. Infection among male participants was significantly higher than among females (OR: 1.7; 95% CI: 1.4–2.0; *P*<0.001). Participants who defecated in latrines were infected significantly less than those who did not (OR: 0.6; 95% CI: 0.4–0.8; *P* = 0.001). Strongyloidiasis cases would be reduced by 39% if all participants defecated in latrines. Incidence of *S. stercoralis* infections did not show a strong tendency toward spatial clustering in this province. The risk of infection significantly decreased with increasing rainfall and soil organic carbon content, and increased in areas with rice fields.

**Conclusions/Significance:**

Prevalence of *S. stercoralis* in rural Cambodia is very high and school-aged children and adults over 45 years were the most at risk for infection. Lack of access to adequate treatment for chronic uncomplicated strongyloidiasis is an urgent issue in Cambodia. We would expect to see similar prevalence rates elsewhere in Southeast Asia and other tropical resource poor countries.

## Author Summary

Data on the prevalence and distribution of *Strongyloides stercoralis* (threadworm) is scarce in many resource-poor countries. We carried out a cross-sectional study during the dry season among 2,396 rural Cambodians of all ages. We used a rigorous diagnostic approach, involving two stool samples per person and two examination techniques, namely, Koga agar plate culture and the Baermann method. We predicted the spatial distribution of *S. stercoralis* using Bayesian Kriging analysis. Almost half of the participants (44.7%) were infected with *S. stercoralis*. Of the *S. stercoralis* cases, 39.7% involved participants under 16 years old. *S. stercoralis* infection prevalence was significantly higher in males than in females. Participants younger than 10 years old had a lower risk of infection than did older participants. Furthermore, our study showed that toilet use could prevent threadworm infections by 39%. Infection prevalence in the province was negatively associated with rainfall and soil organic content and positively associated with land covered by rice fields. We conclude that access to adequate treatment for *S. stercoralis* must be addressed in Cambodia. Infection prevalence is likely to be similar in other countries of the region and the developing world.

## Introduction

*Strongyloides stercoralis,* a soil-transmitted nematode, is a neglected tropical helminthiasis [1], [2] and endemic in tropical, subtropical and temperate settings where sanitary and hygiene conditions are poor [3], [4]. However, the worldwide prevalence of *S. stercoralis* is heterogeneously distributed [2] and the current estimation of infection remains underestimated due to the use of inadequate diagnostic method [5].The available information about *S. stercoralis* infection in developing countries mostly comes from studies in Brazil and Thailand [2].

The gastrointestinal symptoms of the disease include diarrhea and abdominal pain, while dermatological symptoms include itching, rash (urticaria) and migrating larvae in the skin (larva currens) [6]–[9]. However, more than 50% of all infections remain asymptomatic [4]. Due to its particular ability for autoinfection, *S. stercoralis* is the only soil-transmitted helminth (STH) that can lead to systemic infection with high parasite densities and severe to potentially fatal complications, especially in immunosuppressed hosts [3], [7], [10]. Ivermectin is recommended as the most effective treatment [11].

The presence of *S. stercoralis* larvae in stool specimens is proof of infection [12]. Koga-agar plate (KAP) culture [13] and the Baermann method [14] are specific diagnostic methods for strongyloidiasis. However, their sensitivity is not satisfactory when testing a single stool sample in cases of chronic, uncomplicated strongyloidiasis [15]–[18].

In Cambodia, data from several cross-sectional studies in community and hospital settings revealed *S. stercoralis* prevalences between 2.6% and 31.5%. However, in all but three studies, a diagnostic approach with low sensitivity was used on a single stool sample [19]–[22]. Three recent studies used a combined diagnostic approach (KAP culture and Baermann technique) on two [9], [23] and three stool samples [16].

We aimed to determine the prevalence, risk factors and spatial distribution of *S. stercoralis* infection in Preah Vihear province. We conducted a cross-sectional study of *S. stercoralis* infection, using KAP culture and the Baermann method on two stool samples from each participant in 60 villages of Preah Vihear province, northern Cambodia.

## Materials and Methods

### Ethical considerations

The research was approved by the Ethics Committee of the Cantons of Basel-Stadt and Baselland (EKBB, #16/10, dated 1 February 2010), Switzerland, and by the National Ethics Committee for Health Research, Ministry of Health, Cambodia (NECHR, #004, dated 5 February 2010). Written informed consent was obtained from each participant prior to the start of the study. For participants between the ages of 1 and 18 years, written informed consent was obtained from the parents, legal guardian or appropriate literate substitute. All participants were informed of the study's purpose and procedures prior to enrolment.

All participants infected with *S. stercoralis* were treated with a single oral dose of ivermectin (200 µg/kg BW) [24]. All other parasitic infections were treated according to the guidelines of the National Helminth Control Program of Cambodia [25].

### Study setting and population

**Figure 1:**
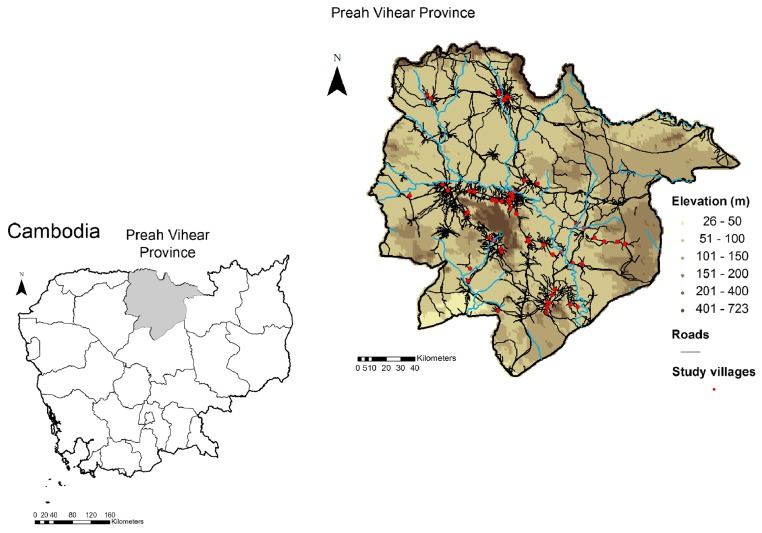
Map of the study villages in Preah Vihear province, Cambodia, 2010.

The study was conducted in 60 rural villages of Preah Vihear province, Northern Cambodia (Figure 1). The villages were randomly selected from a list of all villages in six of the seven districts in Preah Vihear province (total number of villages: 184). The district of Chhaeb was not included as most villages in this district are difficult to access by car, which was necessary to ensure the rapid transfer (three hours by car) of stool samples to one of the two temporary laboratories established in the health centers of Kulen and Rovieng districts.

A cross-sectional study was carried out from February to June 2010 among all the population living in 60 villages. Fifteen households were randomly selected from the list of all households in the selected villages. All household members one year of age and older were eligible for inclusion in the study and all household members present on the day of the survey were enrolled.

### Field and laboratory procedures

After obtaining written informed consent from participants, an individual questionnaire was administered to obtain demographic information (age, gender, educational level and profession), personal risk-perception (knowledge about worm infections), and behavioral data (personal hygiene practices, wearing shoes, and latrine use). The head of household was interviewed, based on a household questionnaire, about socioeconomic indicators such as house type, household assets, latrine and livestock. All questionnaires were pre-tested. After the interview, each participant was given a pre-labeled plastic container (ID code, name, sex, age and date) for stool sample collection. The next morning, the filled stool container was collected and a second empty, pre-labeled one was handed out for a second stool sample of the following day.

Stool samples were transported at ambient temperature and arrived at the laboratory within three hours of collection. Laboratory technicians from the National Center for Parasitology, Entomology and Malaria Control (CNM), Phnom Penh, processed the stool specimens in one of two laboratories established in Kulen and Rovieng health centers, respectively. First, a single Kato-Katz thick smear [26] was prepared using the WHO standard template and examined under a light microscope to detect helminth eggs. Eggs were counted and recorded for each helminth species separately. Second, KAP culture [13] was used to detect *S. stercoralis* larvae. A hazelnut-sized stool sample was placed in the middle of the agar plate and the closed Petri dish was incubated in a humid chamber for 48 hours at 28°C. Afterwards, the plates were visually examined for the presence of larval tracks. The plates were then rinsed with sodium acetate-acetic acid-formalin (SAF) solution. The eluent was centrifuged and the sediment was examined under a microscope for the presence of larvae. Based on morphology, larvae were identified (i.e., size of buccal cavity, presence of genital primordium (L1), presence of forked tail-end (L3)) as either *S. stercoralis* or hookworm larvae. Finally, the Baermann technique [14] was performed to detect *S. stercoralis* larvae. A walnut-sized stool sample was placed on gauze inserted into a glass funnel and covered with water. The apparatus was exposed for two hours to artificial light directed from below. After centrifuging the collected liquid, the sediment was examined under a microscope for the presence of *S. stercoralis* larvae.

### Quality control

For quality control, the technicians were specifically trained on the morphological criteria for distinguishing hookworm and *S. stercoralis* larvae. Throughout the study period, technicians were rigorously supervised by a qualified microscopist from the Swiss Tropical and Public Health Institute (Swiss TPH), Basel, Switzerland. Any unclear diagnosis was immediately discussed with both the qualified microscopist and the study supervisor.

### Environmental data collection

Day and night land surface temperature (LST), enhanced vegetation index (EVI) and land use/land cover (LULC) were extracted at 1×1 km resolution from Moderate Resolution Imaging Spectroradiometer (MODIS) Land Processes Distributed Active Archive Center (LP DAAC), U.S. Geological Survey (USGS) Earth Resources Observation and Science (EROS) Center (http://lpdaac.usgs.gov). Rainfall estimates (RFE) at 0.1 degree (about 10×11 km) resolution were obtained from the National Oceanic and Atmospheric Administration's (NOAA) Climate Prediction Center (CPC) Famine Early Warning System (FEWS) Rainfall Estimates South Asia, version 2.0 (http://www.cpc.ncep.noaa.gov/products/fews/SASIA/rfe.shtml). Digital elevation data at a resolution of 90×90 m were retrieved from the NASA Shuttle Radar Topographic Mission's (SRTM) Consortium for Spatial Information of the Consultative Group for International Agricultural Research (CGIAR-CSI) database. Soil type data at a spatial resolution of 9×9 km, including bulk density, soil organic carbon content and pH, was extracted from the International Soil Reference and Information Center's (ISRIC) World Inventory Soil Emission Potentials (WISE), version 1.0 (http://www.isric.org). The 18 land cover type 1 classes (IGBP) were merged into five categories according to similarity and respective frequencies. Yearly means, as well as minima and maxima of EVI, monthly LST and RFE were calculated for May 2009 to April 2010.

### Statistical analyses

#### Questionnaire and laboratory data

Questionnaire and laboratory data collected from each participant were entered twice and validated in EpiData version 3.1 (EpiData Association; Odense, Denmark). Statistical analyses were performed with STATA version 12.1 (StataCorp.; College Station, TX, USA). Only participants with complete records (two stool samples examined with all diagnostic methods and completed questionnaires) were included in the final analyses. Smoothed age prevalence was used to present the infection prevalence distribution by participant's age. *P-values* less than 0.05 were considered to indicate a significant association.

Data on household assets and livestock were used to build the socioeconomic status (SES), employing principle component analysis (PCA). SES status was defined according to one of three wealth tiers, from poor to least poor [27].

Generalized Estimating Equations (GEE) were used to assess the association between infection status and demographic variables, hygienic status, knowledge, recent medical history of participants and environmental factors. Variables with odds ratios below 0.80 and above 1.25 in the bivariate models were selected for inclusion in the multivariate GEE model. Population attributable fraction was calculated for significantly associated risks.

#### Environmental data

ArcGIS version 10.0 (ESRI; Redlands, CA, USA) was used for environmental data processing, geo-referencing and map making. Environmental data was linked to parasitological and questionnaire data according to location. Data management and bivariate regressions were performed in STATA version 12.1. Bayesian multivariate models were fitted using WinBUGS version 1.4.3 (Imperial College & Medical Research Council; London, UK). Spatial analysis was performed using mixed logistic regression models. The association between infection risk and environmental covariates was assessed at a 15% significance level, as determined by the likelihood ratio test (LRT), with mixed bivariate logistic regressions accounting for village-level correlation with an exchangeable random effect. To explore the village-level correlation of *S. stercoralis* infection risk, Bayesian mixed logistic models were run in absence of covariates. Bayesian models with or without environmental covariates were run alternately with a spatial and a non-spatial exchangeable random effect. Spatial models assumed a stationary isotropic process, with village-specific random effects following a normal distribution with mean zero and a variance-covariance matrix being an exponential function of the distance between pairs of locations. Non-informative prior distributions were chosen for all other parameters. Markov chain Monte Carlo (MCMC) simulation was used to estimate model parameters [28]. Convergence was assessed by examining the ergodic averages of selected parameters. For all models, a burn-in of 5,000 was followed by 50,000 iterations, after which convergence was reached. Results were withdrawn for the last 10,000 iterations of each chain, with a thinning of 10. Model fit was appraised with the Deviance Information Criterion (DIC). A lower DIC indicates a better model [29]. DIC was retrieved after 10,000 additional iterations.

*S. stercoralis* infection risk was predicted at non-surveyed locations using Bayesian Kriging [30]. For model validation, 48 randomly selected villages were used for fitting and the 12 remaining were used as test locations. Models were run with either a spatial random effect, using the WinBUGS “spatial.unipred” function, or with an exchangeable random effect [31]. Predictive ability was assessed using the probability coverage of the shortest Bayesian credible interval, the Mean Squared Error and a χ^2^ test analogue [32], [33]. Based on environmental factors only, predictions were made at 16,532 pixels of a 1×1 km resolution, using an exchangeable random effect.

## Results

### Compliance and study population

Overall, 3,560 individuals from 616 households (average household size: 5; range: 1–12) were enrolled, of which 2,748 (77.2%) participants submitted two stool samples. The final analysis included 2,396 (67.3%) participants with complete data records, i.e., two stool specimens examined with all diagnostic tests and all questionnaires completed.

The median age of the participants was 20 years, with a range from 1 to 85 years. One thousand three hundred and fifty-five (56.5%) participants were females. Half of the participants (48.5%) were farmers and 33.0% were pupils. The majority of participants (58.3%) had attended primary school; one third (32.2%) had not received primary education.

### Parasitological findings

Seven intestinal parasite species were found in the stool samples. Hookworm and *S. stercoralis* were most common, with a prevalence of 46.7% and 44.7%, respectively. *Taenia* sp. was found in 0.4% of participants, while *Hymenolepis nana* and *Enterobius vermicularis* were observed in 0.2% and 0.1% of participants, respectively. Both *Ascaris lumbricoides* and *Trichuris trichiura* were observed in 0.3% of participants. Of the 1,071 *S. stercoralis* cases, 642 (59.9%) were co-infected with hookworm.

### Performance of the diagnostic methods

Table 1 summarizes the results of KAP culture and Baermann tests for the 1,071 *S. stercoralis* cases (44.7%) detected. KAP culture and the Baermann technique detected 877 and 823 cases, respectively. The total of all positive cases diagnosed by any of the two methods was considered the “diagnostic gold standard”. The sensitivity of the KAP culture was 81.9%, and that of the Baermann technique, 76.8%. The negative predictive values were 87.2% and 84.2%, while the positive predictive values were 81.8% and 76.8% for KAP culture and Baermann technique, respectively.

**Table 1:** Koga agar plate (KAP) culture and Baermann method for the detection of *S. stercoralis* in 2,396 participants, Preah Vihear province, Cambodia, 2010.

		Combined Methods (KAP culture and Baermann)		Total
		Positive	Negative	
	**Positive**	877	0	877
**KAP culture**	**Negative**	194	1,325	1,519
	**Total**	1,071	1,325	2,396
	**Positive**	823	0	823
**Baermann Technique**	**Negative**	248	1,325	1,573
	**Total**	1,071	1,325	2,396

### Characteristics of *Strongyloides stercoralis* cases

**Figure 2:**
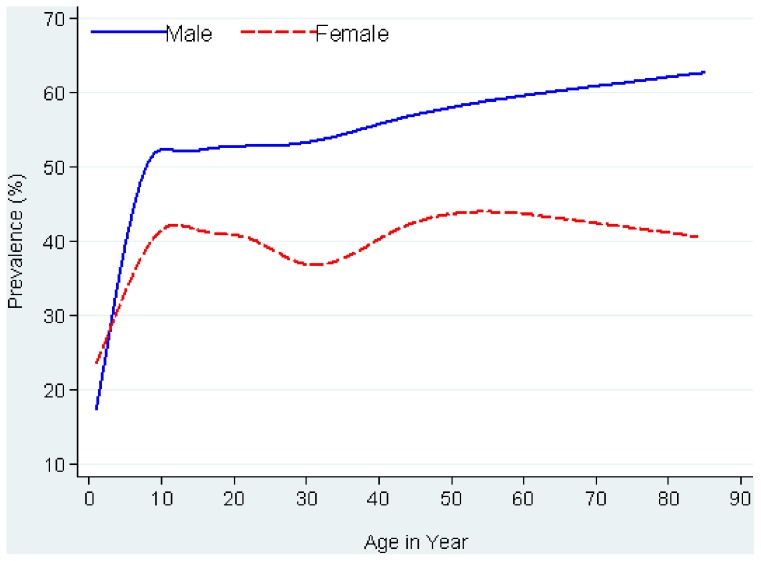
Smoothed age prevalence of *S. stercoralis* infection by sex among 2,396 participants in Preah Vihear province, Cambodia, 2010.

Of 1,071 *S. stercoralis* cases, half were females (50.1%), half were farmers (51.1%), and 425 (39.7%) cases were diagnosed in individuals under 16 years. The majority (57.0%) attended primary school, while one third (33.6%) reported no schooling. Figure 2 shows the smoothed age prevalence stratified by gender. The prevalence of *S. stercoralis* increased rapidly with age, particularly in the first eight years of life, where after it leveled off in females but continued to rise slowly in males. Prevalence rose from 31.4% in children, aged five, to 51.2% in participants older than 50. In all age groups, prevalence was higher in males than in females.

The multivariate GEE found that gender was significantly associated with *S. stercoralis* infection (mOR: 1.7; 95% CI: 1.4–2.0; *P*<0.001). Compared to children under six years old, all age groups had a higher risk for infection. Participants who reported having been treated for worms were less frequently infected with *S. stercoralis* than those who did not report taking anthelminthic drugs (mOR: 0.7; 95% CI: 0.6–0.8; *P*<0.001). In addition, participants who usually defecated in latrines were significantly less infected with *S. stercoralis* than those who did not use latrines (mOR: 0.6; 95% CI: 0.4–0.8; *P* = 0.001). No additional predictor of *S. stercoralis* infection relating to personal disease perception and hygiene was found in the multiple regression analysis. Looking at environmental factors, risk significantly decreased with increasing rainfall (mOR: 0.8; 95% CI: 0.7–0.9; *P* = 0.004) and soil organic carbon content (mOR: 0.6; 95% CI: 0.5–0.9; *P* = 0.003). The land cover class corresponding to croplands was associated with an increased risk for infection (mOR: 1.7, 95%CI: 1.2–2.4; *P* = 0.004) (Table 2).

**Table 2:** Risk factors for *S. stercoralis* infection in the multivariate GEE among 2,396 participants, Preah Vihear province, Cambodia, 2010.

	*S. stercoralis* Negative	*S. stercoralis* Positive	mOR (95% CI)	p-Value
	(N = 1325)	(N = 1071)		
	n (%)	n (%)		
**Gender** (men compared to women)	507 (38.37)	534 (49.9)	1.7 (1.4–2.0)	<0.001
**Age group**				
1–5 years	129 (9.7)	59 (5.5)	Reference	
6–15 years	453 (34.2)	366 (34.2)	2.3 (1.4–3.6)	<0.001
16–30 years	352 (26.6)	287 (26.8)	1.8 (1.2–3.0)	0.01
31–45 years	220 (16.6)	174 (16.2)	1.7 (1.0–2.8)	0.04
>45 years	171 (12.9)	185 (17.3)	2.2 (1.4–3.7)	0.001
**Profession**				
Farmer/Rice-Grower	615 (46.4)	547 (51.1)	Reference	
Schoolchildren	451 (34.0)	339 (31.6)	0.8 (0.6–1.2)	0.275
Others	259 (19.6)	185 (17.3)	1.0 (0.7–1.3)	0.859
Has been treated for worms (yes)	450 (33.9)	279 (26.0)	0.7 (0.6–0.8)	<0.001
Knows about worms/infection with worms (yes)	240 (18.1)	234 (21.8)	1.3 (1.1–1.7)	0.017
Usually defecates in toilet (yes)	195 (14.7)	81 (7.5)	0.6 (0.4–0.8)	0.001
Has shoes (yes)	1203 (90.8)	999 (93.3)	1.1 (0.8–1.6)	0.657
Night temperature (year mean)	-	-	0.9 (0.8–1.1)	0.359
Rainfall (year mean)	-	-	0.8 (0.7–0.9)	0.004
Soil organic carbon content (10–20 g/kg)	773 (58.3)	485 (45.3)	0.6 (0.5–0.9)	0.003
**Land cover**				
Savanna and shrubland	441 (33.3)	283 (26.4)	Reference	
Forest	155 (11.7)	130 (12.1)	1.3 (0.8–2.1)	0.233
Grassland	114 (8.6)	70 (6.6)	1.4 (0.8–2.4)	0.315
Cropland and crop-natural vegetation mosaic	615 (46.4)	588 (54.9)	1.7 (1.2–2.4)	0.004

mOR: Multiple Odds Ratio; 95% CI: 95% Confidence Interval.

During the two weeks preceding examinations for *S. stercoralis*, 50.5% of participants reported an episode of diarrhea, 12.7% had experienced nausea and 59.1% complained about abdominal pain. However, none of these clinical symptoms was significantly associated with *S. stercoralis* infection.

Population attributable risk analysis found that the number of strongyloidiasis cases would be reduced by 39% if all participants used a latrine for defecation.

### Spatial analysis of *Strongyloides stercoralis* infection risk

The spatial model run without covariates indicated very little spatial correlation of infection risk, as indicated by the 1 km range. The small residual (unexplained) within village variance (σ) also indicated a weak clustering tendency of *S. stercoralis* infection risk. Parameters of these models are presented in Table 3. After introducing LST night, rainfall, soil carbon content and land cover, the model with an exchangeable random effect fitted the data slightly better, as indicated by the lower DIC. Environmental covariates explained 45% of the village-level variability and the range dropped under a kilometer after covariates were introduced in the model.

**Table 3:** Model parameters in absence or presence of covariates for the spatial models and their non-spatial counterparts.

	No covariates				Environmental covariates			
Model parameters	non spatial		spatial		non spatial		spatial	
	Median	95% CI	Median	95% CI	Median	95% CI	Median	95% CI
DIC	3136.4		3135.6		3130.6		3130.8	
Σ	0.4	(0.2–0.7)	0.4	(0.2–0.7)	0.2	(0.1–0.4)	0.2	(0.1–0.4)
Ρ	n.a.	n.a.	317.8	(23.6–1558.0)	n.a.	n.a.	812.5	(98.8–1594.0)
Range (km)	n.a.	n.a.	1.1	(0.2–14.0)	n.a.	n.a.	0.4	(0.2–3.2)

CI, credible interval; DIC, deviance information criterion (a measure of model fit; a lower DIC indicates a better fit).

σ is the location-specific unexplained variance.

ρ is the decay parameter. The range (range = 3/ρ) is the distance at which the spatial correlation becomes less than 5%.

### Prediction of *Strongyloides stercoralis* infection risk and model validation

Mixed bivariate logistic regressions revealed no association at 15% significance level between *S. stercoralis* infection risk and any yearly summary measure of altitude, LST day, EVI, soil pH or bulk density. LST night (*P* = 0.072), yearly means of rainfall estimates (*P*<0.0001), soil organic carbon content (*P* = 0.002) and land cover (*P* = 0.107) were associated with infection risk and were used to predict *S. stercoralis* infection risk throughout Preah Vihear province. Apart from LST night, all covariates remained significant in the multivariate model and ORs were similar to those obtained in the multivariate GEE for the risk factor analysis (data not shown). Maps of the covariates used predict infection in Preah Vihear province are presented in Figure S1.

**Figure 3:**
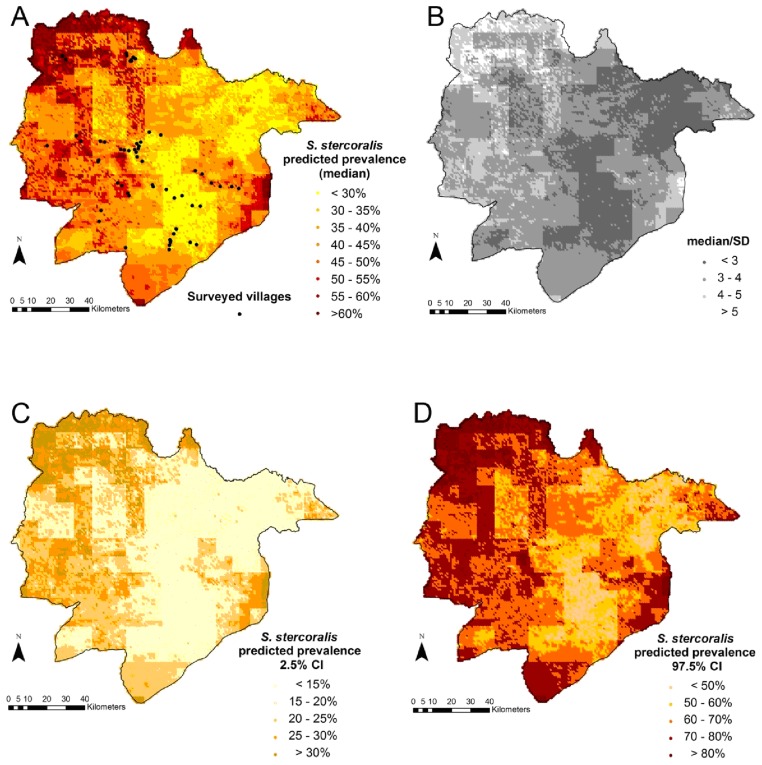
Predicted *S. stercoralis* median prevalence (a), error coefficient (b); lower (c), and higher (d) estimates of *S. stercoralis* predicted prevalence in Preah Vihear province, Cambodia. Legend: The error coefficient is the ratio of the predicted median over its standard deviation; a higher value indicates a higher precision. The lower and upper estimates correspond to the 2.5% and 97.5% borders of the Bayesian credible interval, respectively.

Model validation revealed that both models were able to correctly predict prevalence for 100% of the test locations, within a 95% credible interval. However the non-spatial model, i.e. with an exchangeable random effect, had slightly better predictive ability (MSE: 0.0226 and 0.0229, χ^2^: 13.22 and 13.59 for the non-spatial and spatial models, respectively). Therefore, the non-spatial model was used to predict *S. stercoralis* infection risk in Preah Vihear province, Cambodia. Figure 3 displays the *S. stercoralis* predicted median prevalence in Preah Vihear province (Figure 3A), together with the uncertainty of the estimates (Figure 3B) as expressed by the error coefficient (the ratio between the predicted median and its standard deviation). The lower (2.5%) and upper (97.5%) credible intervals of the predicted *S. stercoralis* prevalence are presented in Figure (3C) and (3D), respectively. Results were consistent with observed prevalence at surveyed locations.

## Discussion

Many epidemiological aspects of *S. stercoralis* infection are poorly understood [34]. The available information on the prevalence of *S. stercoralis* comes from studies on other STHs, where diagnostic methods with low-sensitivity for *S. stercoralis* and only a single stool sample were mostly used [1], [2], [16]. To reach an acceptable estimate of the “true” prevalence of *S. stercoralis*, Siddiqui and Beck [12], and Khieu et al. [16] proposed analyzing multiple stool samples with multiple diagnostic techniques simultaneously.

In our study of *S. stercoralis* among a rural population living in 60 villages in northern Cambodia, we examined two stool samples using two diagnostic techniques (KAP culture and Baermann method) specifically targeting *S. stercoralis* and found that 44.7% of the participants were infected. Children under the age of six accounted for 5.5% of the infections, while prevalence increased with age.

Almost every second individual in our study population was infected with *S. stercoralis*. To our knowledge, this is one of the highest prevalence ever reported, compared to other studies in highly endemic areas like Cambodia [2], [16], [35], Laos [36], Thailand [37], Brazil [34] and China [38], or in other countries. The main reason for such high prevalence is likely to be due to the more rigorous diagnostic approach employed in our study (number of stool specimen, multiple diagnostic methods), compared to the other studies, where a single method to examine a single fecal sample was used. Yet, the prevalence we observed is also substantially higher than that of other studies using the similar diagnostic approaches. Two recent studies in Kandal and Takeo provinces in Cambodia reported that about a quarter (24.4%) of schoolchildren and 21.0% of the general population were infected, respectively [16], [35]; while Steinmann et al., and Knopp et al. found a prevalence rate of 11.7% in a village in Yunnan, China and of 10.8% among schoolchildren in Zanzibar, respectively [39], [40]. Hence, other factors such socioeconomic and sanitary conditions are likely to contribute to the differences observed.

In the absence of a gold standard for diagnosing *S. stercoralis*, KAP culture [13] and the Baermann method [14] are widely used for detecting the parasite microscopically. Our study found that KAP culture was more sensitive than the Baermann method, which is consistent with reports from Cambodia [16], [35], rural Côte d′Ivoire [41], Brazil [42] and Honduras [43]. However, the opposite was observed in studies in south-central Côte d′Ivoire [44], Zanzibar [40], China [39] and Uganda [45]. This seems to indicate that neither method is superior. As either technique will fail to identify a certain number of infections, the combined use of both methods is recommended for optimal sensitivity.

We found that about one third of children under six (59 of 188 children) were already infected with *S. stercoralis*. This hints at a high contamination of the environment, such that children easily become infected when playing on the ground around the house or barefoot in the village. The fact that prevalence steadily increases with age can be explained by the fact that once infected at a young age, an infection can persist in an untreated individual for their entire life [46], [47]. Personal hygiene (not using a toilet for defecating) as a significant predictor of *S. stercoralis* infection was also observed in a study in south-central Cambodia [16]. This connection is obvious: with proper disposal of the feces, contamination of the surrounding area with infective larvae decreases. We calculated that 39.0% of *S. stercoralis* cases in the study area could be prevented if everyone were to defecate in a toilet. The cycle of *S. stercoralis* transmission could thus be interrupted by improving personal hygiene and sanitation. Strongyloidiasis is almost non-existent in countries where sanitation and human waste disposal have improved [48].

*S. stercoralis* infections were ubiquitous the study setting and exhibited a weak tendency to spatial clustering in the Preah Vihear province, as indicated by the low location-specific variance parameter. A low clustering tendency was also observed for hookworm, in the Region of Man, Côte d′Ivoire and Ghana [49], [50]. However, the lack of spatial correlation in this analysis is likely due to the study's small scale. This does not preclude *S. stercoralis* infection risk from spatially clustering at country or regional level, since environmental factors delimit suitable ecological zones for parasites at larger scales. [51].

Still, even at this provincial scale, we found significant associations with rainfall, soil organic carbon content and croplands both in the predictive model and after adjusting for demographic and behavioral factors. Our risk predictions yielded two broad risk zones: a lower risk zone in the East of the province and a higher risk zone in the West, characterized by lower rainfall and soil organic carbon content and a higher proportion of zones occupied by cropland. Since there was no indication of spatial correlation, risk prediction was carried out using an exchangeable random effect and relied on the predictors only. While a negative association between rainfall and infection risk was also identified in Thailand, a laboratory study found that *S. stercoralis* development was impaired by submersion of stools in water [52]. Hypothetically, the decreased risk of *S. stercoralis* infection in the East of the Province where rainfall was higher, might relate to more extensive or long lasting flooding that could negatively affect *S. stercoralis* transmission. Another possibility might be that higher rainfall in the East reduces parasite survival rates, as parasites are washed away by run-off water down steeper slopes. We found that lower soil carbon content was associated with increased risk of infection (in the West). A full profile of soil type information was unavailable for this setting and soil organic carbon content depends on a complex interplay of environmental and soil features, so interpretation is limited. But, in general, soil organic content tends to decrease with increased forest destruction, burning of savannas and land use for agriculture [53]. Hence, the association of increased risk of infection with lower soil carbon contents in our setting might relate to human activities such as slash-and-burn practices that destroy forests to create agricultural lands. Moreover, risk of infection was found to increase in croplands, a MODIS land cover category that specifically corresponds to soils that are alternately bare and cultivated. In our setting, these are rice fields [54]. Half (51.7%) of the study villages are surrounded by rice fields and 54.9% of participants infected with *S. stercoralis* live in such environments. Risk might be increased further by regular soil contamination by defecation around the fields and exposure during agricultural activities. Indeed, open defecation was the usual habit for 88.5% of participants. Finally, the small cluster size (1 km) of infection risk suggests that *S. stercoralis* transmission occurs within villages rather than between them and may relate to the location of defecation sites within and close to the villages.

We conclude that *S. stercoralis* infection is highly prevalent in rural communities of Cambodia. School-aged children and adults over 45 years were the most at risk for infection. Almost 40% of infections could be avoided by proper personal hygiene. Access to adequate treatment for chronic uncomplicated strongyloidiasis is low. Given its potential to produce potentially fatal disseminated infections, further epidemiological data on this parasite in other endemic areas are urgently needed

## Supporting Information

Figure S1 Distribution of environmental factors used to predict *S. stercoralis* prevalence in Preah Vihear province, Cambodia.
(TIF)

Text S1 Results of bivariate risk analysis for *S. stercoralis* infection.
(DOCX)
