# Supplementary material for: High Prevalence and Spatial Distribution of Strongyloides stercoralis in Rural Cambodia
Source: PLoS Negl Trop Dis. 2014 Jun 12;8(6):e2854. doi: 10.1371/journal.pntd.0002854 (PMC4055527; doi:10.1371/journal.pntd.0002854)
Supplement: Text S1 — Results of bivariate risk analysis for S. stercoralis infection. (DOCX) [file pntd.0002854.s002.docx]

**Supporting Text S1: Results of bivariate risk analysis for *S. stercoralis* infection**

Association of demographic, socio-economic, behavioral and environmental factors with *S. stercoralis* infection in bivariate GEEs among 2396 participants in Preah Vihear province, Cambodia, 2010

|  | **Non-*S. stercoralis* (N=1325)** | ***S. stercoralis* (N=1071)** | **OR (95% CI)** | **p-Value** |
| --- | --- | --- | --- | --- |
|  | **n (%)** | **n (%)** |  |  |
| **DEMOGRAPHIC INFORMATION** |  |  |  |  |
| Gender (male) | 507 (38.37) | 534 (49.9) | 1.6 (1.4 – 1.9) | <0.001 |
| Age group |  |  |  |  |
| 1 - 5 years | 129 (9.7) | 59 (5.5) | Reference |  |
| 6 - 15 years | 453 (34.2) | 366 (34.2) | 1.9 (1.4 - 2.7) | <0.001 |
| 16 - 30 years | 352 (26.6) | 287 (26.8) | 1.9 (1.4 - 2.7) | <0.001 |
| 31 - 45 years | 220 (16.6) | 174 (16.2) | 1.9 (1.3 - 2.7) | 0.001 |
| > 45 years | 171 (12.9) | 185 (17.3) | 2.5 (1.7 - 3.6) | <0.001 |
| Profession |  |  |  |  |
| Farmer/Rice-Grower | 615 (46.4) | 547 (51.1) | Reference |  |
| Pupil | 451 (34.0) | 339 (31.6) | 0.9 (0.7 - 1.1) | 0.179 |
| Others | 259 (19.6) | 185 (17.3) | 0.8 (0.6 – 1.0) | 0.02 |
| Education level |  |  |  |  |
| No school | 413 (31.2) | 360 (33.6) | Reference |  |
| Primary school | 786 (59.3) | 610 (57.0) | 1.1 (0.9 - 1.3) | 0.355 |
| Secondary school/High school | 126 (9.5) | 101 (9.4) | 1.1 (0.8 - 1.5) | 0.571 |
| **PERSONAL DISEASE PERCEPTION** |  |  |  |  |
| Have been treated for worms (yes) | 450 (33.9) | 279 (26.0) | 0.7 (0.6 - 0.8) | <0.001 |
| Know about worms/infection with worms (yes) | 240 (18.1) | 234 (21.8) | 1.5 (1.2 - 1.8) | 0.001 |
| Know how to get infected with Worm (yes) | 112 (8.4) | 78 (7.3) | 0.9 (0.7 - 1.2) | 0.456 |
| Know health problem because of Worm (yes) | 168 (12.7) | 150 (14.0) | 1.2 (1.0 - 1.6) | 0.11 |
| **PERSONAL HYGIENE** |  |  |  |  |
| Toilet at home (yes) | 190 (14.3) | 81 (7.6) | 0.6 (0.4 - 0.8) | 0.001 |
| Usually defecated in toilet (yes) | 195 (14.7) | 81 (7.6) | 0.6 (0.4 - 0.8) | <0.001 |
| Washed hand after defecating (yes) | 975 (73.6) | 777 (72.6) | 1.0 (0.8 - 1.16) | 0.685 |
| Washed hand before eating (yes) | 1226 (92.5) | 990 (92.4) | 1.1 (0.8 - 1.5) | 0.611 |
| Wash hand usually with Soap/Ash (yes) | 410 (30.9) | 322 (30.1) | 1.1 (0.9 - 1.3) | 0.49 |
| Had shoes (yes) | 1203 (90.8) | 999 (93.3) | 1.4 (1.0 - 1.9) | 0.026 |
| Socio-economic status |  |  |  |  |
| Poor | 403 (30.4) | 330 (30.8) | Reference |  |
| Less poor | 412 (31.1) | 362 (33.8) | 1.1 (0.9 - 1.4) | 0.364 |
| Least poor | 510 (38.5) | 379 (35.4) | 1.0 (0.8 - 1.2) | 0.722 |
| **ENVIRONMENTAL FACTORS** |  |  |  |  |
| Night Land Surface Temperature, year mean |  |  | 0.9 (0.7 - 1.0) | 0.061 |
| Rainfall, year mean |  |  | 0.73 (0.6 - 0.9) | <0.001 |
| Soil organic carbon content |  |  | 0.6 (0.4 - 0.8) | 0.001 |
| Land Use/Land cover |  |  |  |  |
| Savanna and shrubland | 441 (33.3) | 283 (26.4) | Reference |  |
| Forest | 155 (11.7) | 130 (12.1) | 1.3 (0.8 - 2.3) | 0.319 |
| Grassland | 114 (8.6) | 70 (6.6) | 1.0 (0.5 – 2.0) | 0.959 |
| Cropland and crop-natural vegetation mosaic | 615 (46.4) | 588 (54.9) | 1.5 (1.1 - 2.3) | 0.025 |
| OR: Odd Ratio; 95%CI: 95% Confidence Interval |  |  |  |  |
